# Functional Genome Prediction and Genome-Scale Metabolic Modeling of the Rhizobacteria *Serratia liquefaciens* Strain UNJFSC002

**DOI:** 10.3390/genes17020169

**Published:** 2026-01-30

**Authors:** Cristina Karina Andrade Alvarado, Zoila Felipa Honorio Durand, Sergio Eduardo Contreras-Liza, Gianmarco Castillo, William Andres Guzman Sanchez, Diego Hiroshi Takei-Idiaquez, Julio E. Ballen-Gavidia, Carlos I. Arbizu, Pedro M. Rodriguez-Grados

**Affiliations:** 1 Departamento de Agronomía, Universidad Nacional José Faustino Sánchez Carrión (UNJFSC), Lima 15136, Peru; candrade@unjfsc.edu.pe (C.K.A.A.); scontreras@unjfsc.edu.pe (S.E.C.-L.); 2 Facultad de Bromatologia y Nutricion, Universidad Nacional Jose Faustino Sanchez Carrion (UNJFSC), Lima 15136, Peru; zhonorio@unjfsc.edu.pe; 3 Facultad de Ciencias, Universidad Nacional Jose Faustino Sanchez Carrion (UNJFSC), Lima 15136, Peru; 1735172006@unjfsc.edu.pe (G.C.); wguzman@unjfsc.edu.pe (W.A.G.S.); idiaquez200@gmail.com (D.H.T.-I.); ballenj159@gmail.com (J.E.B.-G.); 4 Facultad de Ingeniería y Ciencias Agrarias, Universidad Nacional Toribio Rodríguez de Mendoza de Amazonas (UNTRM), Amazonas 01001, Peru; 5 Centro de Investigación en Germoplasma Vegetal y Mejoramiento Genético de Plantas (CIGEMP), Universidad Nacional Toribio Rodríguez de Mendoza de Amazonas (UNTRM), Amazonas 01001, Peru

**Keywords:** NGS, phenotyping, genomics, microbiology, metabolic and system biology

## Abstract

Background/Objectives: *Serratia liquefaciens* is a bacterium commonly found in the rhizosphere and may possess PGPR capabilities. The present study aimed to elucidate the genomic, phylogenomic, and metabolic characteristics of *S. liquefaciens* strain UNJFSC002 to determine whether it is an effective PGPR. Methods: The genome of strain UNJFSC002 was obtained from NCBI and annotated using Prokka. Functional genome prediction, phylogenetic reconstruction, and comparative genomics were performed using bioinformatics tools. A GEM model was reconstructed to simulate metabolic fluxes associated with nitrogen fixation, phosphate solubilization, and phytohormone biosynthesis. Computational phenotyping and in silico functional validation were also performed. Results: The draft genome (5.19 Mb, GC 55.33%) contained 4792 protein-coding genes, 4 rRNAs, and 81 tRNAs, with 100% completeness. ANI and core genome phylogeny confirmed its taxonomic position within *S. liquefaciens*, with an identity higher than 98.8%. Pangenome analysis of 25 *Serratia* genomes revealed an open and highly dynamic pangenome (30,515 orthologous groups), indicating extensive genetic plasticity. Functional annotation identified key genes associated with nitrogen and phosphate acquisition, as well as the biosynthesis of IAA and GABA, findings that were supported by GEM simulations, reinforcing its potential as a biofertilizer. Conclusions: The genomic approach confirmed that strain UNJFSC002 harbors multiple active genes and metabolic pathways associated with plant growth promotion and environmental resilience.

## 1. Introduction

The use of plant-growth-promoting rhizobacteria (PGPR) has attracted increasing interest in agriculture and horticulture due to their ability to stimulate plant development, low cost, and ease of application in crops. These bacteria, which naturally inhabit the rhizosphere near plant roots, provide a sustainable alternative to reduce dependence on agrochemicals and mitigate soil contamination. Their isolation and application as biofertilizers allow the beneficial properties of these microorganisms to be harnessed, optimizing plant growth and productivity [1,2].

Various species of PGPR contribute significantly to plant development through multiple mechanisms. Among these are biological nitrogen fixation, solubilization of inorganic phosphates, transformation of essential nutrients, and the production of bioactive compounds such as phytohormones (auxins, gibberellins), siderophores, as well as the enzyme ACC deaminase, polyamines, and ethanolamines. These metabolites enhance the availability of nitrogen and other nutrients for the plant. To achieve this efficiency, PGPR have developed specialized metabolic pathways and a complex regulatory network that controls intracellular nitrogen levels at both the transcriptional and post-transcriptional levels [3,4,5]. It has also been demonstrated that many bacteria possess an active polyamine metabolism, including species from the genera *Bacillus* spp., *Streptomyces* spp., *Azospirillum* spp., among others [6]. These bacterial traits are of vital importance for enhancing plant growth, productivity, and phytosanitary status [7].

PGPR bacteria are widely distributed among various bacterial genera, with the most representative being *Azoarcus*, *Azospirillum*, *Bacillus*, *Herbaspirillum*, *Enterobacter*, *Gluconacetobacter*, *Streptomyces*, *Pseudomonas*, and *Serratia* [8,9]. These microorganisms are associated with various types of plants, mainly in the roots, assisting in several functions such as growth, production of phytohormones and siderophores, also being useful as biofertilizers, and in other cases for bioremediation [10,11]. Over many years, PGPR have generated interest due to their diverse functions. They exhibit chemotaxis properties, as well as antagonism and synergy in relation to plant roots [12]. The use of PGPR inoculants in the plant rhizosphere has been reported as one of the most effective strategies to promote plant development under water stress conditions [13]. Additionally, PGPR have been used for potato growth, regulating its physiological response under water deficit, saline conditions, and reducing the use of NPK fertilizers [14].

*Serratia* is a genus of Gram-negative, facultatively anaerobic bacteria that belong to the Yersiniaceae family [15]. This genus of bacteria is found in a wide variety of habitats, including water, soil, and plants, and can cause diseases in humans [16]. Studies by Zhang [17] have shown that bacteria of the genus *Serratia*, such as *S. marcescens*, have the ability to promote the development of various plant species through the production of indole-3-acetic acid (IAA), which can also significantly enhance root growth. However, the genus *Serratia* has been considered an opportunistic human pathogen, affecting immunocompromised patients [18]. At the same time, this species has been isolated from natural environments such as soils, bodies of water, insects, and plant leaves, including the rhizosphere, where it has the ability to positively influence plant growth promotion [19]. The broad niche and functional gene diversity of this species is important in discussions about the existence and differentiation between nosocomial and non-nosocomial populations, as well as in the detection of genes linked to virulence and antibiotic resistance in strains of this species that could be selected and used as PGPR [20]. Analyses using next-generation sequencing (NGS) have allowed the identification and characterization of specific genes associated with PGPR in species of the genus *Serratia*, highlighting their role in promoting plant growth and improving tolerance to water stress [21]. Additionally, draft genomes of *S. fonticola* have been isolated and sequenced—including AU-P3(3), a strain with PGPR functions. This strain produces indole acetic acid, ammonia, hydrogen cyanide, siderophores, and exhibits activity against pathogenic fungi, such as *Rhizoctonia* [22]. Likewise, draft genomes of *S. marcescens* strains with PGPR traits have been isolated and sequenced, including production of IAA, nitrogen fixation, and phosphate solubilization, among other PGPR characteristics [1,17]. On the other hand, recent studies have successfully isolated three strains of the genus *S. liquefaciens* from endophytes present in tomato and lettuce leaves. These strains, in future research, could demonstrate properties with potential for application in promoting plant growth, standing out as promising plant-associated endophytes [23].

Recent advances in high-throughput sequencing have facilitated access to a large amount of genomic information from various environmental isolates. This, together with the development of genome-scale metabolic reconstructions (GEMs) and tools such as flux balance analysis (FBA) [24,25], allows for detailed comparisons of metabolism and transport processes from a systems biology perspective. Moreover, genome-scale metabolic models have become key tools in the study of microbial metabolism, offering a comprehensive platform that allows exploration of how organisms adapt to different environments from a systemic perspective. This approach is especially valuable for simulating conditions that would be difficult or impossible to replicate experimentally [26]. On the other hand, systems biology approaches have contributed to a greater understanding of metabolic pathways and have led to nearly complete metabolic networks for a range of important organisms, from bacteria and yeasts to humans [27]. There are various mathematical approaches to analyze these networks [25]. These include stochastic models, deterministic models, and Boolean models applied to predict the biological system of an organism [28,29]. Each of these approaches offers particular advantages depending on the type of system and the level of detail required. Considering that in systems biology, the comprehensive understanding of complex organisms such as bacteria is often approached through two complementary methods, top-down and bottom-up [30], these two approaches are currently revolutionizing microbiome engineering by reducing costs and enabling new transformations. Sciences such as synthetic biology and applied biotechnology in the design of microbial consortia have been key points in the use of these methodological approaches [31,32,33].

In this study, we present an investigation of the *S. liquefaciens* strain UNJFSC002 strain, a cultivable microorganism with potential as a PGPR. Unlike other reported species of the *Serratia* genus, this bacterium exhibits plant-beneficial traits; however, its genomic, phylogenetic, and functional characterization remains limited, representing a significant gap in the study of pathogenic PGPR. The complete genome of the strain, previously sequenced and available in GenBank, allowed for ANI and phylogenetic analyses specifically with strains of the same genus, assessing its genetic identity and evolutionary relationships. Additionally, computational phenotyping was used to predict metabolic functions associated with plant growth promotion. These approaches were integrated with biochemical assays and the reconstruction of a genome-scale metabolic model (GEMs), providing a comprehensive characterization of the strain’s functional capabilities and highlighting its potential for sustainable agricultural applications.

## 2. Materials and Methods

### 2.1. Genomic Data Collection and Assembly

The genomic sequencing data of *S. liquefaciens* strain UNJFSC002 were obtained from the National Center for Biotechnology Information (NCBI) (SRA SRR28705404, BioSample SAMN39932967, and BioProject PRJNA1076138). According to the isolation data, it was obtained from the rhizosphere of a potato crop (cv. Bicentenaria) in an experimental field at the Universidad Nacional José Faustino Sánchez Carrión (UNJFSC) in Lima, Peru [34]. Furthermore, the study by Andrade [35] demonstrated the field-level plant-growth-promoting capacity of *S. liquefaciens* strain UNJFSC002 in potato production, as well as its in vitro abilities for IAA production, biological nitrogen fixation, and moderate phosphate solubilization of tricalcium phosphate. Therefore, this study focuses on verifying the metabolic pathways underlying these functions. Read quality was assessed using FastQC v0.12.1 [36]. Additionally, reads were trimmed and filtered (Q-28) using Trimmomatic v0.36 [37] and fastp v0.20.1 [38] with default parameters.

### 2.2. Genome Assembly, Annotation and Functional Analysis

To obtain the genome of *S. liquefaciens* strain UNJFSC002, a de novo assembly was performed using the Unicycler v0.5.0 assembler [39]. This assembly algorithm was selected due to its diverse methodological approaches, which enable optimization of the assembly with SPAdes, resulting in longer contigs used for this genome. Afterwards, QUAST v5.2.0 [40] was used to evaluate the assembly statistics. Subsequently, the assembly was evaluated using BUSCO v5.2.2 [41] to assess the completeness of our genome assembly in comparison with three other strains deposited in the NCBI Datasets Genome. The script is available at (https://github.com/GianmarcoCastillo/Serratia-liquefaciens-strain-UNJFSC002, accessed on 3 June 2025).

Additionally, the Microbial Genome Atlas (MiGA) v2.0 [42] was used to assess genome quality, contamination, and identification. Genome annotation was performed using Prokka [43] and RAST [44]. The Barrnap v0.9 tool [43] was used for rapid prediction of ribosomal RNA, and ARAGORN [45] was employed for rapid tRNA prediction. To complement the functional genome annotation performed by Prokka, orthology-based tools were employed using the KEGG server, specifically BlastKOALA (KEGG Orthology and Links Annotation) [46]. Subsequently, the KASS annotation suite [47] was used, which employs BLAST v2.16.0 and the BBH (bidirectional best hit) method. These tools enabled the precise assignment of gene functions and the reconstruction of key metabolic pathways of the organism. Additionally, DeepNOG v1.2.2 [48] was applied, a specialized tool for evolutionary analysis, functional annotation, and metabolic pathway modeling, which identifies COGs (Clusters of Orthologous Genes) using alignment methods based on Hidden Markov Models, thereby enhancing the functional and phylogenetic accuracy of the predictions. The annotations were then compared to perform data curation. Finally, GenoVI v0.2.15 [49] was used to automatically visualize the genomic map of strain UNJFSC002, integrating functional information in a graphical format with specific color palettes, facilitating the interpretation and analysis of gene and metabolic pathway distribution within the genome.

### 2.3. Phylogenetic Tree Analysis, Comparative Genome and Pan Genome Analysis

The phylogenetic tree of *S. liquefaciens* strain UNJFSC002 was reconstructed by retrieving a set of 81 core bacterial genes from 29 genomes, including our strain. These genes were aligned using the Up-to-date Bacterial Core Gene (UBCG) Software v2.0 [50]. A maximum likelihood (ML) tree was estimated using IQ-TREE v2.3.5 [51], incorporating ModelFinder [52] to identify the best-fitting substitution model. A total of 1000 bootstrap replicates were generated. Of the 29 genomes analyzed, four *Azotobacter* strains were used to root the phylogenetic tree.

In the genomic comparison and molecular confirmation of *S. liquefaciens* strain UNJFSC002, the complete genome sequence (WGS) was used. An average nucleotide identity (ANI) analysis was conducted between *S. liquefaciens* strain UNJFSC002 and 24 other *Serratia* strains obtained from the NCBI genomic database (Table 1). These strains were selected based on experimental data previously validated in earlier studies. This analysis was performed using the pyANI v0.3.0 [53] and dRep v3.4 [54] tools. Both algorithms were employed to generate a corresponding heat map of the 25 bacterial genomes, allowing visualization of cluster grouping. Additionally, the specific position of our bacterium was identified using the dendrogram generated by dRep, which includes information on ANI percentages. The pangenome analysis of the 25 strains was performed using Roary v3.13.0 [55], utilizing GFF3 files obtained from Prokka. The analysis was conducted with the following parameters: a BLASTP identity threshold of 90%, clustering in groups of 100,000 sequences, and separation of paralogs.

### 2.4. Computational Phenotyping

Computational phenotyping was performed by analyzing the genome sequence of the *S. liquefaciens* strain UNJFSC002 isolate, along with the aforementioned *Serratia* strains, which have demonstrated strong PGPR activity in published studies, as well as two *Azotobacter* and *Azospirillum* strains obtained from NCBI GenBank. After manual curation, the genomes were re-annotated using Prokka [56], characterizing the presence or absence of genes or features related to functional classes of interest regarding their potential as biofertilizers: (1) Nutrient acquisition, (2) Phytohormone biosynthesis, and (3) Antimicrobial resistance (AMR). Nutrient acquisition is divided into the following three subcategories: (1a) nitrogen acquisition (*fnr*, *nar*, *nas*, *nif*, and *nir*), (1b) iron acquisition (*bfr*, *ent*, *exb*, and *fhu*), and (1c) phosphate acquisition (*app*, *gcd*, *phn*, *pho*, *phy*, and *ppa*). Phytohormone biosynthesis is divided into two subcategories: (2a) GABA biosynthesis (*gab*, *gbu*, *prr*, and *puu*) and (2b) IAA biosynthesis (*ald*, *ipd*, *trp*, and *tyr*). The gene groups were selected through a literature search on PubMed at NCBI, focusing on those that had experimental results supporting their involvement in PGPR functions. AMR levels were predicted using Abricate v1.2.0, obtained from GitHub (https://github.com/tseemann/abricate, accessed on 3 June 2025). The results were visualized as heat maps using Seaborn v0.13 [57], Matplotlib v3.10 [58], and Pandas v2.2.3 [59] in Python v3.10.12. The data and results are available on GitHub (https://github.com/GianmarcoCastillo/Serratia-liquefaciens-strain-UNJFSC002, accessed on 3 June 2025).

### 2.5. Genome-Scale Metabolic Models Network S. liquefaciens Cepa UNJFSC002

To reconstruct the model and explore the metabolic capabilities of our strain UNJFSC002, CarveME v1.5.1 [60] was used with default pipeline arguments to process a draft metabolic reconstruction of the strain’s genome. CarveME is a bioinformatics tool that enables the reconstruction of genome-scale metabolic models using a top-down approach to build models for individual bacterial strains and microbial communities, offering speed and high scalability. The bioinformatics tool leverages the BIGG Models database [61] to obtain information on metabolites and reactions (metabolomics and fluxomics), and uses eggNOG-mapper [62] as the primary source for functional orthology annotation. These models behave similarly to manually curated models in reproducing experimental phenotypes, such as gene essentiality and substrate utilization. The annotation performed by Prokka, in .faa format, was input into the CarveME pipeline using the command-line argument ‘$ carve –protein SERRATIA.faa’ for reconstruction, followed by the output file Serratia.xml. The reference template iJO136.xml was used, classifying the bacterium with the ‘-u gramneg’ option, standardizing with ‘–fbc2’, and performing gap-filling for the metabolic model using the ‘–gapfill M9’ option. Additionally, the obtained model was polished using ModelPolisher v2.1 [63]. This polishing process involves moving entries from the notes section to the annotation section of an entity, annotating all entities with their respective BIGG IDs. Furthermore, the polished model was evaluated using the MEMOTE v0.11.1 [64] metabolic package via its command-line version. It was used to compare the model against the standards for model description. Additionally, the Escher v1.8.1 [65] and pyCOBRA v0.29.0 [66] packages were used to visualize the metabolic model network, allowing the representation of fluxes across the different metabolic pathways of the model. Escher enables the construction of metabolic pathways through reactions, metabolites, and genes, providing context for the organism’s metabolism. Meanwhile, pyCOBRA was used for the computational manipulation and simulation of the metabolic model, including flux balance analysis (FBA) to predict metabolites involved in nitrogen fixation, phosphate, IAA, and GABA. The codes used for the respective reconstruction can be found in the following GitHub repository: (https://github.com/GianmarcoCastillo/GEMs-UNJFSC002, accessed on 3 June 2025).

### 2.6. Screening for Plant-Growth-Promoting Traits

In vitro studies of *S. liquefaciens* strain UNJFSC002 were conducted by Alvarado et al. [35]; therefore, these experiments were repeated to provide consistency with our predictive data. Tests included assessing the strain’s ability to solubilize phosphate using NBRIP medium (National Botanical Research Institute’s Phosphate Growth Medium) [67]. Next, IAA production was assessed using a liquid culture medium enriched with 5 mM L-tryptophan as a precursor, following the protocol described by Sánchez [68]. The ability for biological nitrogen fixation was evaluated in a nitrogen-free mineral medium, following the method described by Zúñiga [69]. Finally, the cellulolytic activity of the strain was assessed using culture plates containing carboxymethyl cellulose (CMC) agar as the carbon source, following the method proposed by Samira [70]. All these tests were performed in triplicate, using a control strain from the laboratory as a control.

## 3. Results

### 3.1. Genomic Data Collection and Assembly of S. liquefaciens Strain UNJFSC002

The data obtained for *S. liquefaciens* strain UNJFSC002 yielded a total of 11,235,041 raw reads, with an average length of 153 base pairs (bps), a GC content of 55.33%, and a total sequencing output of 1.7 gigabytes (GBs).

**Figure 1:**
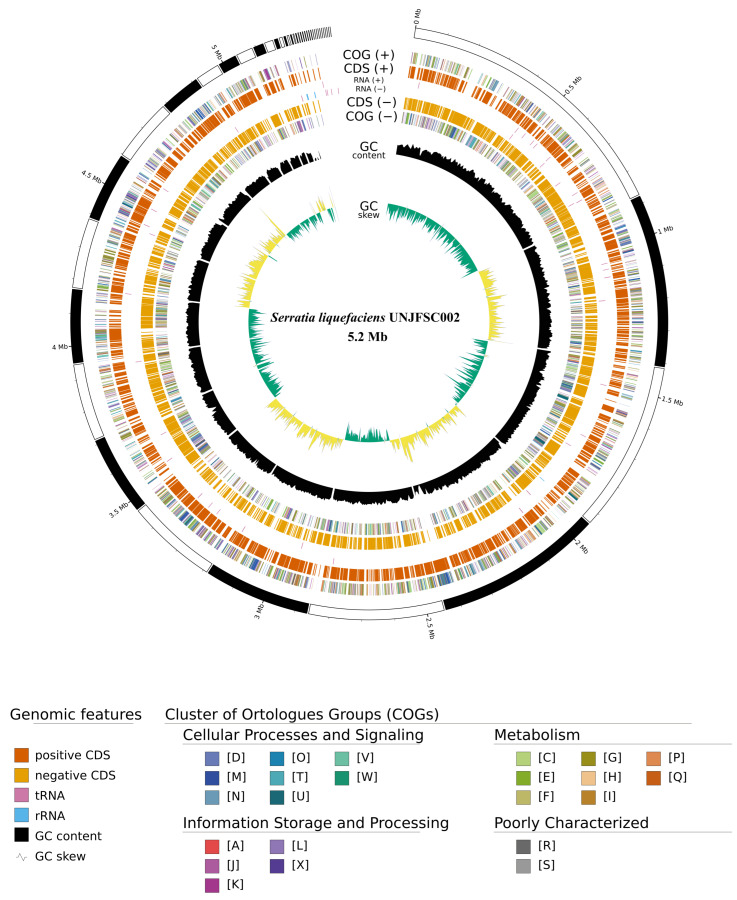
Genomic map of *S. liquefaciens* strain UNJFSC002. Protein-coding sequences (both + and − strands) are shown in orange, tRNAs in pink, rRNAs in light blue, GC content is represented in black, and finally, GC Skew+ and GC Skew- are displayed in dark green and light green, respectively. The COGs shown in gray represent general functions, cellular processes and signaling are indicated in sky blue and olive green (categories D, M, N, O, T, U, V, W), metabolism is depicted in light green, dark green, and orange (categories C, E, F, G, H, I, P, Q), and information storage and processing are colored in red and purple (categories A, J, K, L, and X).

The de novo assembly was performed with Unicycler optimized with SPAdes, producing improved N50 values and better contigs by testing different k-mers ranging from 27 to 127. QUAST results yielded a statistical value of 44, with a GC content of 55.33%. The longest contig obtained was 5,197,394 bp (≥1000 bp). Additionally, the N50 value was 414,860 bp and the L50 was 5. In the taxonomic classification analysis of strain UNJFSC002 using MiGA, the results indicate that the strain belongs to the genus *Serratia* (*p*-value: 0.0015) and is likely to belong to the species *S. liquefaciens* (*p*-value: 0.018). The closest relative in the database was *S. liquefaciens* FDAARGOS_125 (NZ_CP014017), with an ANI of 98.97%. Additionally, MiGA provided other metrics such as completeness, contamination, and quality, reporting a completeness of 100%, contamination of 1.9%, and a quality score of 90.5 (Table 2). Finally, genome annotation yielded the following results: a total genome size of 5,199,434 bp, 4792 protein-coding sequences, and predictions by Barrnap and ARAGORN identified 4 rRNAs, 1 tmRNA, and 81 tRNAs (Figure 1).

**Figure 2:**
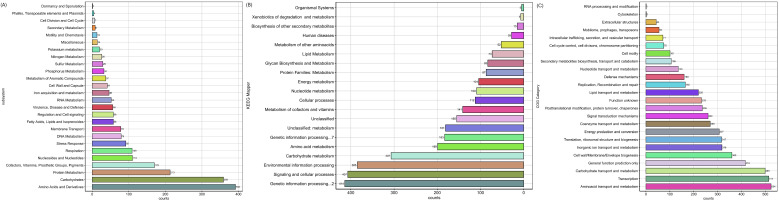
Functional annotation of genes and proteins in *S. liquefaciens* strain UNJFSC002 was performed to determine their biological functions. The analysis included: (**A**) identification of the SEDD functional domain, (**B**) pathway mapping using KEGG Mapper, and (**C**) classification into functional categories according to COG (Clusters of Orthologous Genes).

Furthermore, based on the genomic map results obtained with GENOVI, 80 of the 81 tRNAs have a GC content between >53% and ≤55.43%, while the remaining tRNA has a GC content below 50%. Additionally, only one of the rRNAs has a GC content of 56.02%, while the rest have a GC content below 50%. Moreover, the first 18 contigs display a GC content ranging from over 53.57% to 56.02%. On the other hand, subsystem analysis identified genes involved in nitrogen metabolism, totaling 26, genes involved in phosphorus metabolism, totaling 32, proteins related to general metabolism, totaling 213, amino acids and amino acid derivatives, totaling 392, and cofactors, vitamins, and prosthetic groups, totaling 170 (Figure 2A). KEGG Mapper analysis identified various metabolic pathways and cellular processes associated with the genes in the bacterial genome under study as follows: secondary metabolite biosynthesis, totaling 52, signaling and cellular processes, totaling 407, energy metabolism, totaling 104, cofactor and vitamin metabolism, totaling 141, protein families involved in metabolites, totaling 87, and 155 elements that could not be assigned to specific functional categories in KEGG (Figure 2B).

Finally, in the COG category results, 524 genes were identified as involved in amino acid transport and metabolism, 514 genes related to transcription, 106 genes associated with the biosynthesis, transport, and catabolism of secondary metabolites, 307 genes involved in energy production and conversion, 269 genes related to coenzyme transport and metabolism, 318 genes involved in inorganic ion transport and metabolism, and 233 genes with functions yet to be determined (Figure 2C).

### 3.2. Comparative Analysis of the Complete Genome and Phylogeny

**Figure 3:**
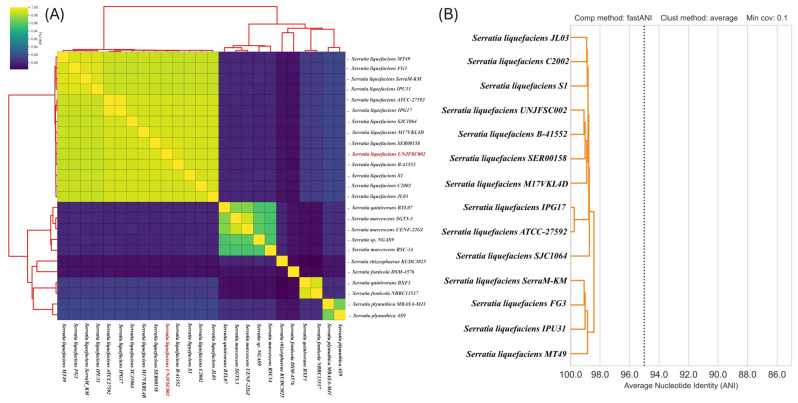
(**A**) Heatmap of the ANI among the 25 bacterial strains of the genus *Serratia*. (**B**) Dendrogram of the cluster where our strain UNJFSC002 is located among the other strains of the genus *Serratia*.

The comparative analysis of the complete genome based on ANI of 25 individual strains, different from *Serratia*, showed that they were divided into four groups. Two strains, *S. fonticola* DSM-4576 and *S. rhizosphaerae* KUDC3025, did not belong to any group (Figure 3A).

Furthermore, the group containing our strain was composed of 15 *Serratia* strains, with strain UNJFSC002 being closely related to three *S. liquefaciens* strains B-41552 (98.8 ANI), SER00158 (98.7 ANI), and M17VKL4D (98.4 ANI), as shown in the dendrogram in Figure 3B. This high ANI value supports the classification of UNJFSC002 within the species *S. liquefaciens*, as it exceeds the generally accepted threshold of 95–96% for bacterial species delineation [71].

**Figure 4:**
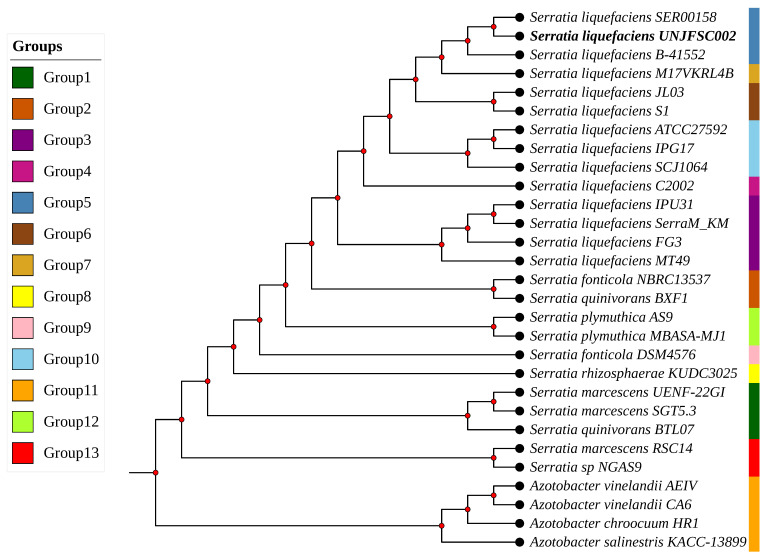
Phylogenetic tree of 25 strains of the genus *Serratia* and 4 strains of the genus *Azotobacter*, the tree inference was constructed using 81 core genes.

A phylogenetic tree was then constructed based on 81 bacterial core genes obtained from the UBCG2 database, with the aim of analyzing the evolutionary relationship between our strain and the 28 selected genomes. These genes are suitable for phylogenetic reconstructions in bacteria, as they are highly conserved and perform essential (COG) functions in most bacterial lineages. Moreover, they provide a more accurate and reliable evolutionary resolution than phylogeny based solely on the 16S rRNA gene [50,72]. Among the genomes included were 25 bacteria of the genus *Serratia* and four additional strains of the genus *Azotobacter*, all retrieved from the NCBI database. Thirteen distinct groups were identified, as shown in Figure 4.

The analyzed strains were distributed into thirteen well-defined phylogenetic clades, showing clear evolutionary differentiation within the genus *Serratia*. Group 1 consisted of *S. marcescens* UENF-22GI, *S. marcescens* SGT5.3, and *S. marcescens* BTL07; Group 2 included *S. fonticola* NBRC13537 and *S. quinivorans* BXF1; while Group 3 comprised *S. liquefaciens* MT49, *S. liquefaciens* SerraM_KM, *S. liquefaciens* IPU31, and *S. liquefaciens* FG3. Group 4 was represented solely by *S. liquefaciens* C2002, while Group 5 included *S. liquefaciens* strain UNJFSC002 the focus of this study together with *S. liquefaciens* B-41552 and *S. liquefaciens* SER00158. Group 6 included *S. liquefaciens* S1 and *S. liquefaciens* JL03, while Group 7 was composed exclusively of *S. liquefaciens* M17VKRL4B. Meanwhile, Groups 8 and 9 were represented by individual strains: *S. rhizosphaerae* KUDC3025 and *S. fonticola* DSM4576, respectively. Group 10 comprised *S. liquefaciens* SJC1064, *S. liquefaciens* IPG17, and *S. liquefaciens* ATCC27592; Group 11 included *A. chroococcum* HR1, *A. vinelandii* CA6, *A. vinelandii* AEIV, and *A. salinestris* KACC_13899; Group 12 encompassed *S. plymuthica* AS9 and *S. plymuthica* MBASA-MJ1; and finally, Group 13 consisted of *S. marcescens* RSC-14 and *Serratia* sp. NGAS9. In accordance with these results, *S. liquefaciens* strain UNJFSC002 was located within Group 5, sharing a monophyletic clade with *S. liquefaciens* B-41552 and *S. liquefaciens* SER00158. This phylogenetic grouping is consistent with the results obtained from the ANI analysis, which also places these strains within the same cluster, confirming their close phylogenetic relationship. The high nucleotide identity values (>98%) observed among these strains reinforce their classification within the same species and provide strong evidence of a recent and conserved evolutionary relationship within the *S. liquefaciens* lineage.

### 3.3. Comparative Analysis of the Pangenome of S. liquefaciens UNJFSC002

**Figure 5:**
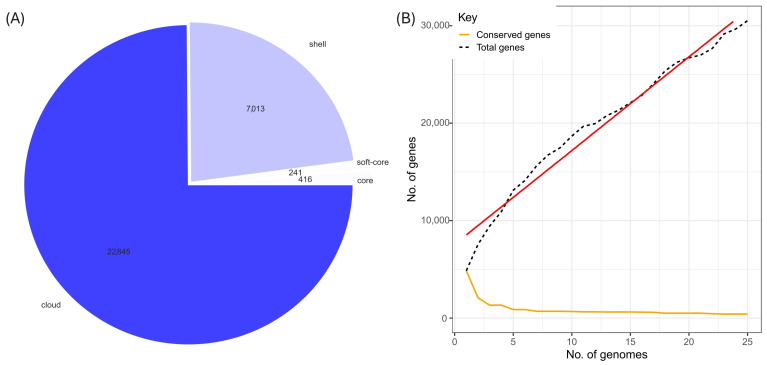
(**A**) Pie chart of the pangenome of the 25 *Serratia* bacterial strains. (**B**) Heap’s law plot of the 25 bacterial strains, with a red trend line indicating the increase in genes as genomes are added, and an orange line showing the reduction in conserved genes.

The pangenome of *S. liquefaciens* strain UNJFSC002 compared against 24 genomes was analyzed and is shown in Figure 5. Core genes encompass essential functions related to cellular maintenance and primary metabolism shared by all strains, whereas accessory and cloud genes include determinants of ecological adaptation and functional specialization. In particular, the latter harbor metabolic pathways associated with plant growth promotion. In this case, a total of 30,515 groups of orthologous genes were identified in the pangenome analysis, of which 416 genes constituted the core genome, 241 corresponded to the soft core, 7013 to the shell genes, and 22,845 to the cloud genes (Figure 5A). This distribution reflects a marked genetic variability among the analyzed strains, suggesting an open pangenome, as confirmed by the application of Heap’s law (Figure 5B). This pattern indicates that the incorporation of new strains would continue to increase the total number of genes, revealing high genomic plasticity and a dynamic adaptive evolution within the genus. The trend of the Heap’s law graph for the pangenome shows a gradual expansion due to the addition of new genomes, with the slope continuing to increase, indicating that the bacterial species in this study has an open pangenome, as shown by the red trend line. The orange curve shows the reduction in the number of conserved genes, suggesting high genetic variability among the analyzed strains. As more genomes are incorporated, it becomes less likely for a gene to be present in all of them, reflecting great diversity and adaptation to different ecological niches (Figure 5B).

### 3.4. Computational Phenotyping

**Figure 6:**
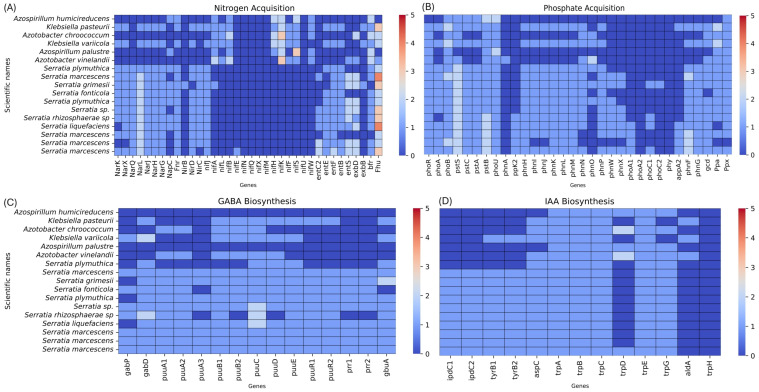
Computational phenotyping of 17 bacteria with PGPR functions, including 11 from the genus *Serratia*, 3 from *Azotobacter*, 2 from *Klebsiella*, and 1 from *Azospirillum*. (**A**) Heatmap of asimilacion de nitrógeno. (**B**) Heatmap of Phosphate Acquisition. (**C**) Heatmap of GABA biosynthesis. (**D**) Heatmap of IAA biosynthesis. Note: Color keys range from 0 (none) to 5 (maximum value).

Computational phenotyping, also known as reverse genomics, was employed to evaluate the potential of the bacterial isolates characterized in this study as PGPR in Peruvian plants. The purpose of this approach was to predict specific phenotypes and genetic or biochemical capabilities of the organisms based on their functionally annotated genomic sequences [73]. This analysis succeeded in identifying bacterial strains that, according to predictions, exhibit a high capacity to promote plant growth while being environmentally friendly [74]. For this purpose, the genomic sequences of the isolates were examined to detect genetic features associated with beneficial functions, such as: (1) Nutrient acquisition, (2) Phytohormone biosynthesis, and (3) antibiotic resistance: these resistance genes are important because they can be transferred to soil strains where the bacterium is applied, potentially passing these genes to the local microbiome (Figure 6 and Table 3).

Our strain UNJFSC0002 presented the highest total number of genes, with the exception of the nitrogen-fixation (*nif*) genes. These genes are present in typical PGPR strains such as *Azospirillum* and *Azotobacter*. This indicates that our strain can assimilate nitrogen but cannot fix it due to the absence of the aforementioned genes.

Regarding the phosphate-solubilization genes, our strain UNJFSC002 and the vast majority of strains possess all the genes except *phnA*, *phnO*, *phoA2*, *phoC3*, *phy*, and *ppa*.

For the genes associated with GABA biosynthesis, these were frequent in UNJFSC002, and notably the *puu* genes, which participate in putrescine degradation and confer tolerance to osmotic/acid stress, thereby enhancing root colonization.

In the case of IAA biosynthesis genes, the strain contained all genes except *trpD*, *aldA*, and *trpH*.

### 3.5. Top-Down Reconstruction Metabolics Model Strain UNJFSC002

**Figure 7:**
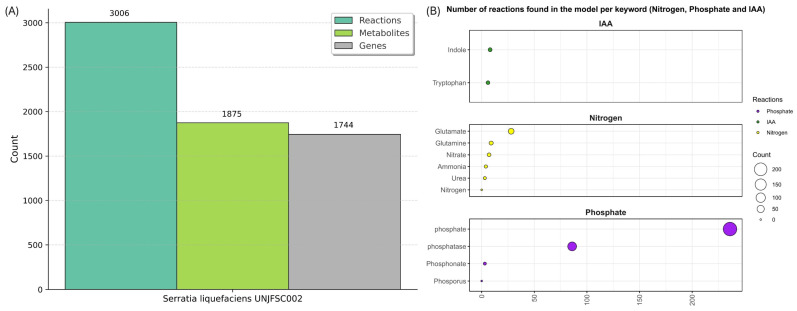
Metabolic model of *S. liquefaciens* strain UNJFSC002 obtained from the iJO136 template (**A**) iGC1744 model for *S. liquefaciens*. (**B**) Reactions found in nitrogen fixation, phosphorus metabolism, and IAA biosynthesis in our metabolic model.

After reconstructing the draft and performing the corresponding polishing with MEMOTE, the development of the model followed an iterative process (Figure 7). The final metabolic model comprised 1875 metabolites, 3006 reactions, and 1744 genes, distributed across three compartments: the cytosol, the periplasm, and the extracellular space. The metabolic model was designated iGC1744, following the nomenclature convention used for genome-scale models, where the letter “i” refers to in silico information, “GC” corresponds to the initials of the model’s author, and the number “1744” represents the total number of incorporated genes [75]. The model comprises 3006 metabolic reactions, whose annotations were compared with various reference databases to validate its functional and metabolic coverage. As a result, 807 matches were found with KEGG [76], 1091 with BioCyc [77], 1759 with ModelSeed [78], 276 with Reactome [79], and 2270 with MetaNetX [80]. No matches were detected with ChEBI [81], suggesting a limited representation of chemical compounds in that database. Likewise, 51 reactions associated with nitrogen fixation, 295 linked to phosphate fixation, 2 related to GABA biosynthesis, and 15 involved in IAA biosynthesis were identified, highlighting the presence of key metabolic pathways involved in microbial functionality and plant-growth-promoting potential.

The reactions involved in nitrogen metabolism are distributed as follows: 28 correspond to glutamate, 9 to glutamine, 3 to urea, 7 to nitrate, and 4 to ammonia; the reactions involved in phosphate metabolism: 236 correspond to phosphate, 86 to phosphatase, and 3 to phosphonate; and the reactions involved in IAA biosynthesis: 6 related to tryptophan and 8 related to indole (Table 4). Moreover, the metabolic network map created using Escher enabled the visualization of metabolic pathways, allowing the identification of complete and incomplete pathways. Although the reactions involved in IAA and GABA biosynthesis could not be visualized, reactions related to nitrogen and phosphorus metabolism were clearly observed. This model, developed using Python, was refined until all errors were eliminated, resulting in the final visualization map containing all the required SBML fields (Figure 7).

### 3.6. Characterization of Strain PGPR

The results of the in vitro determination of the PGPR capacity of strain UNJFSC002 are shown in Table 5.

## 4. Discussion

Rhizosphere bacteria are beneficial microorganisms that inhabit the area surrounding plant roots and promote their growth and development, even under stress conditions. Additionally, they contribute to tolerance against biotic and abiotic factors and protect plants from pathogens, strengthening their resilience and sustainability [82,83,84].

Several strains of the genus *Serratia*, isolated from agricultural rhizospheres, have been recognized as PGPR [85]. These bacteria enhance the health and development of their hosts through mechanisms such as IAA production, biological nitrogen fixation, phosphate solubilization, and siderophore synthesis, in addition to exhibiting antagonistic effects against phytopathogenic fungi [7,86].

Despite the overall similarities between strain UNJFSC002 and other PGPR from the genus *Serratia*, such as *S. marcescens* CDP-13, RSC-14, BTL07, and *S. plymuthica* MBSA-MJ1 [21,87,88,89,90,91,92,93], its genomic architecture exhibits distinctive functional features. The co-occurrence of IPA pathway genes for IAA biosynthesis (*ipdC*, *tyrB*, *aspC*), the *phn* cluster for phosphonate utilization, and an expanded repertoire of regulators involved in anaerobic nitrate respiration (*narX*/*narQ*/*narL*) suggest enhanced adaptive plasticity in nutrient-limited soils. Complementaryally, the iGC1744 model revealed a high density of reactions associated with glutamate and glutamine metabolism, indicating potential advantages in nitrogen assimilation and redistribution, a feature rarely described in other Serratia GEMs. However, the reliance on the metabolic framework of *E. coli* iJO1366 [94] introduces limitations in the reconstruction of specific pathways, particularly those associated with IAA biosynthesis and GABA degradation. This dependence may constrain the complete representation of metabolic routes unique to *Serratia*, since some enzymatic steps or genus-specific transporters may not be included in the base model, thereby affecting the accuracy and comprehensiveness of the resulting GEM. Thus, future validations should integrate condition-specific transcriptomics, targeted metabolomics, and reconstructions based on experimental data derived from *S. liquefaciens*, enabling a more precise resolution of metabolic fluxes and the empirical confirmation of the PGPR phenotypes inferred in silico.

The detection of *blaSPR-1*, *oqxB9*, and *bla-C* in *S. liquefaciens* strain UNJFSC002 introduces key considerations for its use as a biofertilizer under biosafety criteria. These genes, associated with resistance to carbapenems, quinolones, phenicols, and β-lactams, could pose a risk if they are linked to mobile genetic elements, as has been documented for *MBLs* and *OqxAB* efflux systems in *Enterobacteriaceae* [95,96]. Although AmpC-type β-lactamases are typically chromosomal, mobile variants can function as environmental reservoirs with the potential for horizontal transfer [97]. Therefore, characterizing their genomic context and potential mobility is essential, as international regulations discourage the use of bioinoculants carrying transferable ARGs. Taken together, the results do not exclude the use of the strain, but they do require a formal risk assessment prior to its field application.

## 5. Conclusions

Genomic and phylogenetic analyses confirmed that *S. liquefaciens* strain UNJFSC002 belongs to the species *S. liquefaciens*, exhibiting high completeness (100%) and strong phylogenetic clustering with reference strains.

Comparative genomics revealed a highly dynamic and open pangenome among *Serratia* species, indicating extensive genetic diversity and ecological adaptability.

The functional genome prediction and the orthology-based annotation identified groups of genes associated with key PGPR mechanisms, such as phosphate acquisition and IAA biosynthesis.

The genome-scale metabolic model reconstructed for strain UNJFSC002 demonstrated efficient metabolic fluxes in essential nutrient cycles and phytohormone production, corroborating its PGPR biochemical traits.

The integration of computational phenotyping, GEM reconstruction, and experimental validation provides a robust systems biology framework for understanding the metabolic potential of rhizobacteria and guiding the rational design of microbial inoculants for sustainable agriculture.

## Figures and Tables

**Table 1:** Reference genomes of 25 *Serratia* bacterial strains from GenBank.

Strain	NCBI Reference Sequence	Country	Source
*S. plymuthica* AS9	NC_015567.1	Sweden	Rhizosphere
*S. liquefaciens* ATCC27592	CP006252.1	Unknown	N/A
*S. liquefaciens* B-41552	NZ_MQMW00000000.1	United States	Ground beef
*S. marcescens* BTL07	NZ_WNKC00000000.1	Bangladesh	*Capsicum annum* rhizoplane
*S. grimesii* BXF1	NZ_LT883155.1	Portugal	Diseased Pinus pinaster
*S. liquefaciens* C2002	NZ_JACAPD010000021.1	Netherlands	Symptomatic mushroom tissue
*S. fonticola* DSM4576	NZ_CP011254.1	Malaysia	Water
*S. liquefaciens* FG3	NZ_CP033893.1	Brazil	Flower of *Stachytarpheta glabra*
*S. liquefaciens* IPG17	NZ_JAQNAS000000000.1	United States	Imported Fresh Produce 14
*S. liquefaciens* IPU31	NZ_JAWQEN000000000.1	United States	N/A
*S. liquefaciens* JL03	NZ_QCYZ00000000.1	China	Cattle
*S. rhizosphaerae* KUDC3025	NZ_CP041764.1	South Korea	Rhizospheric soil
*S. liquefaciens* M17VKL4B	NZ_JAXOWE000000000.1	South Africa	Leaves
*S. plymuthica* MBSA-MJ1	NZ_JADCNO000000000.1	United States	Environment
*S. liquefaciens* MT49	NZ_CP061082.1	United States	Groundwater from well
*S. grimesii* NBRC13537	NZ_BCTT01000040.1	Japan	N/A
*Serratia* sp. NGAS9	NZ_CP047605.1	Germany	Rhizosphere soil
*S. marcescens* RSC-14	NZ_CP012639.1	South Korea	Root of Solanum nigrum
*S. marcescens* S1	NZ_JADKMB000000000.1	Spain	Human disease
*S. liquefaciens* SER00158	NZ_JADTPQ000000000.1	United States	Wound human
*S. liquefaciens* SerraM_KM	NZ_JAHQRD000000000.1	Greece	Sea bream
*S. marcescens* SGT5.3	NZ_JAJFEW000000000.1	United Kingdom	Fruit
*S. liquefaciens* SJC1064	NZ_CAMKUI000000000.1	United States	Bloodstream
*S. marcescens* UENF-22GI	NZ_LIAI00000000.1	Brazil	Vermicompost
*S. liquefaciens* UNJFSC002	NZ_JBAKHJ000000000.1	Peru	Bicentennial potato soil

**Table 2:** Trimming reads and statistics analysis of *S. liquefaciens* UNJFSC002.

Quality Control FastQ	Results
High-quality reads	10,613,744 (99.760%)
Low-quality reads	19,324 (0.182%)
Contaminated reads	336 (0.003%)
Short reads	5852 (0.055%)
Trimmed adaptors	Yes
Completeness	100
Contamination	1.90%
GC%	55.33
No. of BUSCOs ^a^	439/1/1/0
Contigs N50/L50 (bp)	376/7

^a^ BUSCO genes are reported as: complete/duplicated/fragmented/missing.

**Table 3:** Microbial resistance analysis from computational phenotyping.

Name	Gene	Resistance	Product	Reference
*S. plymuthica* AS9	*oqxB11*	Phenicol;Quinolone	multidrug efflux RND transporter permease subunit OqxB11	NG_050429.1
*S. plymuthica* AS9	*qnrB96*	Quinolone	quinolone resistance pentapeptide repeat protein QnrB96	NG_067167.1
*S. plymuthica* AS9	*bla-C*	Beta-Lactam	class C beta-lactamase	NG_047385.1
*S. marcescens* BTL07	*oqxB9*	Phenicol;Quinolone	multidrug efflux RND transporter permease subunit OqxB9	NG_050458.1
*S. marcescens* BTL07	*blaSST-1*	Cephalosporin	cephalosporin-hydrolyzing class C beta-lactamase SST-1	NG_050144.1
*S. marcescens* BTL07	*tet(41)*	Tetracycline	tetracycline efflux MFS transporter Tet(41)	NG_048142.1
*S. marcescens* BTL07	*aac(6’)_Serra*	Aminoglycoside	aminoglycoside 6’-N-acetyltransferase	NG_052196.1
*S. quinivorans* BXF1	*blaSPR-1*	Carbapenem	putative metallo-beta-lactamase SPR-1	NG_052102.1
*K. variicola* DX120E	*blaFONA-1*	Beta-Lactam	class A beta-lactamase FONA-1	NG_049092.1
*K. variicola* DX120E	*oqxB9*	Phenicol;Quinolone	multidrug efflux RND transporter permease subunit OqxB9	NG_050458.1
*K. variicola* DX120E	*oqxA6*	Phenicol;Quinolone	multidrug efflux RND transporter periplasmic adaptor subunit OqxA6	NG_050424.1
*K. variicola* DX120E	*blaLEN-17*	Beta-Lactam	class A beta-lactamase LEN-17	NG_049271.1
*S. plymuthica* MBASA-MJ1	*oqxB17*	Phenicol;Quinolone	multidrug efflux RND transporter permease subunit OqxB17	NG_050435.1
*S. plymuthica* MBASA-MJ1	*bla-C*	Beta-Lactam	class C beta-lactamase	NG_047385.1
*S. plymuthica* MBASA-MJ1	*qnrE1*	Quinolone	quinolone resistance pentapeptide repeat protein QnrE1	NG_054677.1
*K. pasteurii* NG13	*fosA_gen*	Fosfomycin	FosA family fosfomycin resistance glutathione transferase	NG_047885.1
*K. pasteurii* NG13	*blaOXY-4-1*	Beta-Lactam	class A extended-spectrum beta-lactamase OXY-4-1	NG_050613.1
*K. pasteurii* NG13	*oqxA10*	Phenicol;Quinolone	multidrug efflux RND transporter periplasmic adaptor subunit OqxA10	NG_050418.1
*K. pasteurii* NG13	*oqxB20*	Phenicol;Quinolone	multidrug efflux RND transporter permease subunit OqxB20	NG_050439.1
*Serratia* sp. NGAS9	*oqxB30*	Phenicol;Quinolone	multidrug efflux RND transporter permease subunit OqxB30	NG_050450.1
*Serratia* sp. NGAS9	*blaSRT*	Cephalosporin	SRT/SST family class C beta-lactamase	NG_047548.1
*Serratia* sp. NGAS9	*tet(41)*	Tetracycline	tetracycline efflux MFS transporter Tet(41)	NG_048142.1
*Serratia* sp. NGAS9	*aac(6’)_Serra*	Aminoglycoside	aminoglycoside 6’-N-acetyltransferase	NG_052448.1
*S. marcescens* RSC14	*oqxB25*	Phenicol;Quinolone	multidrug efflux RND transporter permease subunit OqxB25	NG_050444.1
*S. marcescens* RSC14	*aac(6’)_Serra*	Aminoglycoside	aminoglycoside 6’-N-acetyltransferase	NG_052468.1
*S. marcescens* RSC14	*tet(41)*	Tetracycline	tetracycline efflux MFS transporter Tet(41)	NG_048142.1
*S. marcescens* RSC14	*blaSST-1*	Cephalosporin	cephalosporin-hydrolyzing class C beta-lactamase SST-1	NG_050144.1
*S. liquefaciens* UNJFSC002	*blaSPR-1*	Carbapenem	putative metallo-beta-lactamase SPR-1	NG_052102.1
*S. liquefaciens* UNJFSC002	*oqxB9*	Phenicol;Quinolone	multidrug efflux RND transporter permease subunit OqxB9	NG_050458.1
*S. liquefaciens* UNJFSC002	*bla-C*	Beta-Lactam	class C beta-lactamase	NG_047385.1
*S. marcescens* SGT5.3	*tet(41)*	Tetracycline	tetracycline efflux MFS transporter Tet(41)	NG_048142.1
*S. marcescens* SGT5.3	*blaSST-1*	Cephalosporin	cephalosporin-hydrolyzing class C beta-lactamase SST-1	NG_050144.1
*S. marcescens* SGT5.3	*aac(6’)_Serra*	Aminoglycoside	aminoglycoside 6’-N-acetyltransferase	NG_052346.1
*S. marcescens* SGT5.3	*oqxB5*	Phenicol;Quinolone	multidrug efflux RND transporter permease subunit OqxB5	NG_050454.1
*S. marcescens* UENF-22GI	*tet(41)*	Tetracycline	tetracycline efflux MFS transporter Tet(41)	NG_048142.1
*S. marcescens* UENF-22GI	*blaSST-1*	Cephalosporin	cephalosporin-hydrolyzing class C beta-lactamase SST-1	NG_050144.1
*S. marcescens* UENF-22GI	*oqxB25*	Phenicol;Quinolone	multidrug efflux RND transporter permease subunit OqxB25	NG_050444.1
*S. marcescens* UENF-22GI	*aac(6’)-Ial*	Aminoglycoside	aminoglycoside N-acetyltransferase AAC(6’)-Ial	NG_047281.1
*K. pasteurii* NG13	*fosA_gen*	Fosfomycin	FosA family fosfomycin resistance glutathione transferase	NG_047885.1
*K. pasteurii* NG13	*blaOXY-4-1*	Beta-Lactam	class A extended-spectrum beta-lactamase OXY-4-1	NG_050613.1
*K. pasteurii* NG13	*oqxA10*	Phenicol;Quinolone	multidrug efflux RND transporter periplasmic adaptor subunit OqxA10	NG_050418.1
*K. pasteurii* NG13	*oqxB20*	Phenicol;Quinolone	multidrug efflux RND transporter permease subunit OqxB20	NG_050439.1
*K. variicola* DX120E	*oqxB9*	Phenicol;Quinolone	multidrug efflux RND transporter permease subunit OqxB9	NG_050458.1
*K. variicola* DX120E	*oqxA6*	Phenicol;Quinolone	multidrug efflux RND transporter periplasmic adaptor subunit OqxA6	NG_050424.1
*K. variicola* DX120E	*blaLEN-17*	Beta-Lactam	class A beta-lactamase LEN-17	NG_049271.1

**Table 4:** Number of reactions and total flux by keyword (Nitrogen, Phosphate and IAA).

Keyword	Reaction Count	Total Flux	Category
Ammonia	4	318.312751554756	Nitrogen
Nitrate	7	0	Nitrogen
Urea	3	0	Nitrogen
Glutamine	9	246.955109937283	Nitrogen
Glutamate	28	969.168784682527	Nitrogen
Nitrogen	0	0	Nitrogen
Phosphate	236	555.116982396018	Phosphate
Phosphatase	86	196.634440032285	Phosphate
Phosphonate	3	0	Phosphate
Phosphorus	0	0	Phosphate
Indole	8	111.681921901678	IAA
Tryptophan	9	111.681921901678	IAA

**Table 5:** In vitro growth-promoting capacity of *S. liquefaciens* strain UNJFSC002.

In Vitro Test	*S. liquefaciens* UNJFSC002
Phosphate solubilization	+
IAA production	+
Biological nitrogen fixation	−
Cellulolytic activity	−

Note: (+) Positive reaction, (−) Negative reaction.

## Data Availability

The sequence reads generated in this study have been submitted to the Sequence Read Archive (SRA) under the accession number SRR28705977. The assembled genome has been deposited to NCBI GenBank under the accession number JBAKHK000000000, the BioProject accession number PRJNA1076189 and the Biosample accession number SAMN39934077. The PROKKA annotation files have been published on Zenodo (DOI: https://doi.org/10.5281/zenodo.11069170).
